# Supplementary material for: The interactome of intact mitochondria by cross-linking mass spectrometry provides evidence for coexisting respiratory supercomplexes
Source: Mol Cell Proteomics. 2017 Dec 8;17(2):216–32. doi: 10.1074/mcp.RA117.000470 (PMC5795388; doi:10.1074/mcp.RA117.000470)
Supplement: Supplemental Data [file supp_RA117.000470_133922_0_supp_23978_fzffwf.zip › spectra_annotation/mito_spectra_annotation/264-1-5-1-10-1.pdf]

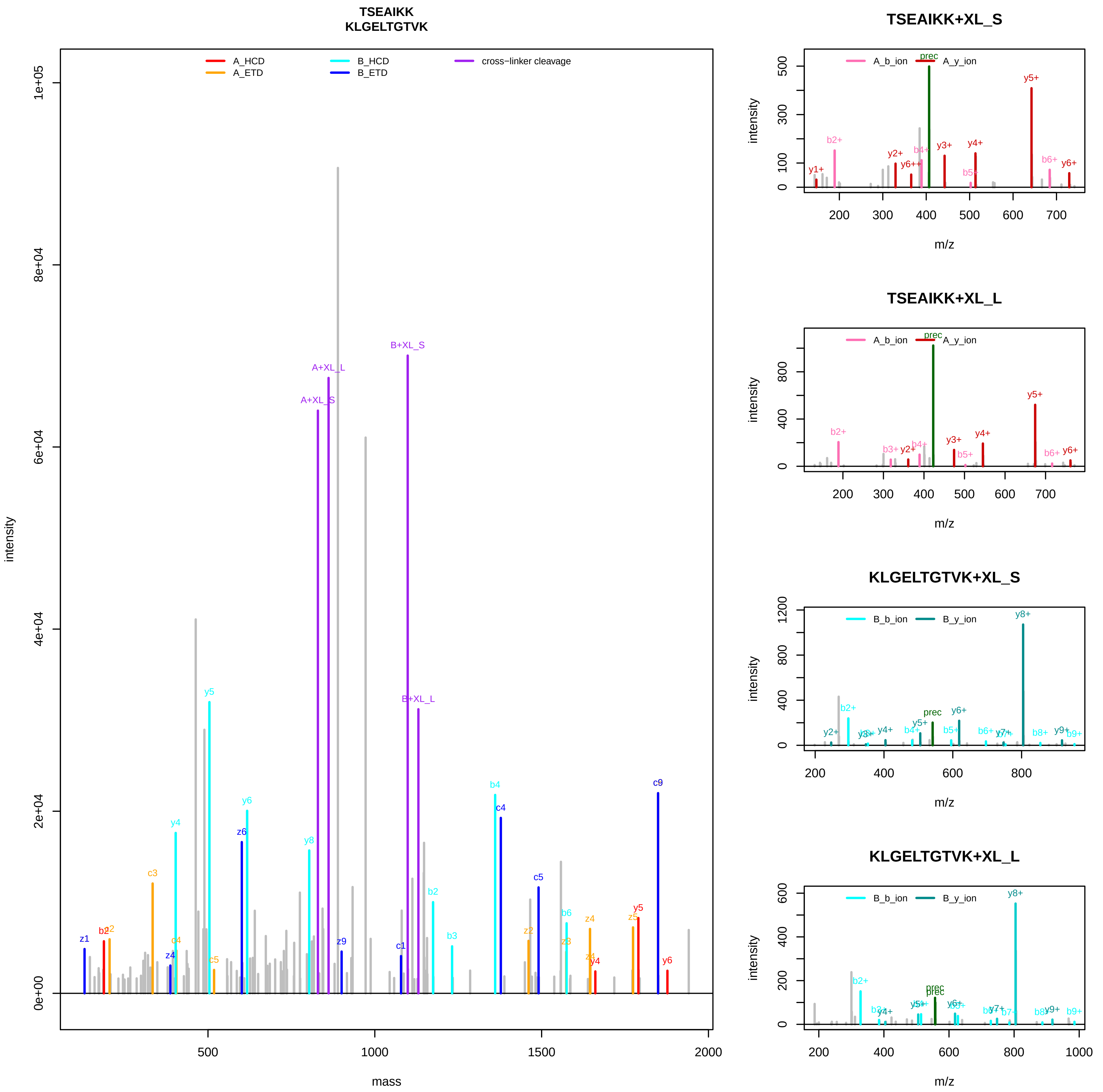
TSEAIKK
KLGELTGTVK
A_HCD
A_ETD
B_HCD
B_ETD
cross−linker cleavage
intensity
mass
0e+00
2e+04
4e+04
6e+04
8e+04
1e+05
500
1000
1500
2000
B+XL_S
A+XL_L
A+XL_S
B+XL_L
y5
y4
y6
z6
y8
c3
z1
b2
z2
c4
z4
c5
z9
c1
b2
b3
b4
c4
c5
b6
z2
z3
z4
y4
z5
y5
c9
y6
TSEAIKK+XL_S
A_b_ion
A_y_ion
prec
intensity
m/z
y1+
b2+
y2+
y6++
b4+
y3+
y4+
b5+
y5+
b6+
y6+
TSEAIKK+XL_L
b3+
KLGELTGTVK+XL_S
B_b_ion
B_y_ion
y2+
y3+
y4+
b4+
y5+
b5+
y6+
b6+
y7+
b8+
y8+
y9+
b9+
KLGELTGTVK+XL_L
b7+
